# Hepatitis of unknown aetiology in children – epidemiological overview of cases reported in Europe, 1 January to 16 June 2022

**DOI:** 10.2807/1560-7917.ES.2022.27.31.2200483

**Published:** 2022-08-04

**Authors:** Adriana Romaní Vidal, Aisling Vaughan, Francesco Innocenti, Soledad Colombe, Lina Nerlander, Natalia Rachwal, Bruno Christian Ciancio, Aikaterini Mougkou, Carlos Carvalho, Enrique Delgado, Piers Mook, Géraldine de Muylder, Michael Peeters, Tencho Tenev, Elitsa Golkocheva-Markova, Veronika Vorobieva Solholm Jensen, Anders Koch, Julie Figoni, Cécile Brouard, Georgia Nikolopoulou, Anastasia Zisouli, Niamh Murphy, Annemarie Broderick, Lital Goldberg, Rivka Rich, Lior Hecht Sagie, Maria Elena Tosti, Barbara Suligoi, Rosa Joosten, Roan Pijnacker, Ingvild Fjeldheim, Eli Heen, Małgorzata Stępień, Piotr Polański, Rui Tato Marinho, João Vieira Martins, Carmen Varela, Ana Avellón, Emmi Andersson, Marie Jansson Mörk, Sema Mandal, Conall Watson, Laura Coughlan, Meera Chand, Claire Neill, Declan T Bradley, Kathy Li, Maureen O’Leary, Neil McInnes, Christopher J Williams, Catherine Moore, Ardiana Gjini, Erika Duffell, Richard Pebody

**Affiliations:** 1 European Centre for Disease Prevention and Control (ECDC), Stockholm, Sweden; 2 World Health Organization Regional Office for Europe, Copenhagen, Denmark; 3 Epidemiology Unit, Regional Health Agency of Tuscany, Florence, Italy; 4 Sciensano, Epidemiology of Infectious Diseases, Brussels, Belgium; 5 Sciensano, Infectious Diseases in Humans, Viral Diseases, National Reference Centre for Hepatitis Viruses, Brussels, Belgium; 6 National Reference Laboratory Hepatitis viruses, NCIPD-Virology, Sofia, Bulgaria; 7 Department of Virus and Microbiological Special Diagnostics, Statens Serum Institut, Copenhagen, Denmark; 8 Infectious Disease Epidemiology and Prevention, Statens Serum Institut, Copenhagen, Denmark; 9 Santé Publique France, the National Public Health Agency, Saint-Maurice, France; 10 Greek National Public Health Organization (EODY), Athens, Greece; 11 Health Service Executive HPSC surveillance scientist on the National IMT for hepatitis, Dublin, Ireland; 12 Children’s Health Ireland (CHI), Crumlin, Ireland; 13 Israel Ministry of Health, Jerusalem, Israel; 14 National Centre for Global Health - Istituto Superiore di Sanità, Rome, Italy; 15 Infectious Disease Department - Istituto Superiore di Sanità, Rome, Italy; 16 National Institute for Public Health and the Environment (RIVM), Centre for Infectious Disease Control, Bilthoven, the Netherlands; 17 Department of Infection Control and Vaccines, Norwegian Institute of Public Health, Oslo, Norway; 18 Department of Epidemiology of Infectious Diseases and Surveillance, National Institute of Public Health NIH – National Research Institute, Warsaw, Poland; 19 Gastroenterology and Hepatology Department, Hospital S. Maria; Medical School of Lisbon; National Programme for Viral Hepatitis, Portugal Ministry of Health, Lisbon, Portugal; 20 Directorate of Information and Analysis, Directorate-General of Health, Lisbon, Portugal; 21 National Centre of Epidemiology, Carlos III Institute of Health, CIBERESP, Madrid, Spain; 22 National Centre of Microbiology, Carlos III Institute of Health, CIBERESP, Madrid, Spain; 23 Public Health Agency of Sweden, Solna, Sweden; 24 United Kingdom Health Security Agency Epidemiology Cell, London, United Kingdom; 25 United Kingdom Health Security Agency Incident Director, London, United Kingdom; 26 Public Health Agency Northern Ireland, Belfast, United Kingdom; 27 Public Health Agency Northern Ireland, Belfast, United Kingdom; 28 Regional Virology Laboratory Belfast Health and Social Care Trust, Northern Ireland, Belfast, United Kingdom; 29 Clinical and Protecting Health Directorate, Public Health Scotland, Glasgow, United Kingdom; 30 West of Scotland Specialist Virology Centre, NHS Greater Glasgow and Clyde, Glasgow, United Kingdom; 31 Public Health Wales, Cardiff, United Kingdom

**Keywords:** hepatitis, adenovirus, unknown aetiology, paediatric acute liver failure, Europe, WHO European Region, TESSy

## Abstract

Following the report of an excess in paediatric cases of severe acute hepatitis of unknown aetiology by the United Kingdom (UK) on 5 April 2022, 427 cases were reported from 20 countries in the World Health Organization European Region to the European Surveillance System TESSy from 1 January 2022 to 16 June 2022. Here, we analysed demographic, epidemiological, clinical and microbiological data available in TESSy. Of the reported cases, 77.3% were 5 years or younger and 53.5% had a positive test for adenovirus, 10.4% had a positive RT-PCR for SARS-CoV-2 and 10.3% were coinfected with both pathogens. Cases with adenovirus infections were significantly more likely to be admitted to intensive care or high-dependency units (OR = 2.11; 95% CI: 1.18–3.74) and transplanted (OR = 3.36; 95% CI: 1.19–9.55) than cases with a negative test result for adenovirus, but this was no longer observed when looking at this association separately between the UK and other countries. Aetiological studies are needed to ascertain if adenovirus plays a role in this possible emergence of hepatitis cases in children and, if confirmed, the mechanisms that could be involved.

## Background

Acute hepatitis refers to a rapid onset inflammation of the liver that can progress to acute liver failure with significant morbidity and mortality. It may affect previously healthy individuals and can be due to several infectious causes, hepatitis A, B and E virus, Epstein-Barr virus and cytomegalovirus being some of the more common, as well as non-infectious causes. In 30-49% of children with acute hepatitis progressing to liver failure, the disease aetiology remains unknown, although this high proportion may be due to the lack of exhaustive diagnostic testing carried out [1-3]. Severe acute hepatitis and acute liver failure of unknown aetiology are not under surveillance in most countries, thus defining a baseline disease incidence is challenging.

## Outbreak detection

An unusual number of severe acute hepatitis cases of unknown aetiology in children was first detected in Scotland and reported by the United Kingdom (UK) to the World Health Organization (WHO) on 5 April 2022 [4,5]. Cases reported by the UK were predominantly between 2 and 5 years-old, otherwise healthy children who presented with jaundice, significantly elevated aspartate or alanine transaminases, while being negative for hepatitis virus A–E [6]. Investigations on possible biological, environmental, toxicological or drug-related aetiologies were inconclusive. The majority (68%) of cases in the UK had a concomitant adenovirus infection. A recent increase in the community circulation of adenovirus infections in children younger than 5 years has also been reported from the UK, which can, however, be partially explained by an increase in testing [7]. Adenovirus infection is therefore one of the main aetiological hypotheses under investigation, although a causal relationship has not been proven and additional cofactors are under investigation.

In addition to an increase in hospital admissions for severe acute hepatitis to specialised liver units in England [8], emergency inpatient admissions and syndromic surveillance data from emergency departments compiled by the UK Health Security Agency (UKHSA) indicated a small rise in relevant diagnoses and emergency room attendances, predominantly in children aged 1-4 years. However data should be interpreted with caution because of the absence of data from before the pandemic and the inclusion of cases with known causes in some analyses [9]. Surveys undertaken in clinical centres across Europe in late April 2022 suggested possible increases in the number of cases or transplants in some centres, but this evidence is weak and limited by small numbers [10,11]. Further investigations using hospital discharge data were still ongoing in June 2022. Following the initial alert from the UK, the European Centre for Disease Prevention and Control (ECDC) and the World Health Organisation (WHO) Regional Office for Europe started joint surveillance of this event in the WHO European Region. 

The objective of this analysis is to describe trends and epidemiological characteristics of cases and identify possible risk factors and aetiological hypotheses to be tested with future analytical studies.

## Methods

### Case definition

We included cases submitted to The European Surveillance System (TESSy) until 16 June 2022 using the ECDC/WHO case definition which includes cases 16 years or younger who have presented since 1 October 2021 with an acute hepatitis (non A–E) with aspartate transaminase or alanine transaminase over 500 IU/L. It excludes cases with a known aetiology such as specific infectious diseases, drug toxicity, metabolic, hereditary or autoimmune disorders [12]. Because of differences in national case definitions, where several countries did not include cases before 1 January 2022, we restricted the analysis to cases with a date of onset of first symptoms of the disease (where available), or a date of hospitalisation, from 1 January to 16 June 2022. Since, in some cases, gastrointestinal and other symptoms can be observed weeks before the acute hepatitis is diagnosed, the reporting protocol specified that the date of onset of symptom that should be reported referred to the one when the first symptom appeared. 

### Data collection and analysis

Demographic, epidemiological, clinical and microbiological data on cases are reported to TESSy. We analysed case distribution by age and sex. We calculated mean and median age for cases except those from Northern Ireland, as they were reported by age group only. We calculated unadjusted odds ratios (OR) and 95% confidence intervals (CI) using Firth logistic regression to assess the associations between age and clinical outcome and between age and positivity for adenovirus and severe acute respiratory syndrome coronavirus 2 (SARS-CoV-2). Similarly, we assessed the associations between positivity for adenovirus or SARS-CoV-2 with admission to an intensive care unit or high dependency unit (ICU/HDU) and with liver transplantations. We compared case characteristics between the UK and other countries in terms of age distribution, prevalence of adenovirus and SARS-CoV-2 and ICU/HDU admission using Pearson's chi-squared test. We also calculated the OR for the association between positivity to adenovirus and reporting country (UK vs other countries), adjusting by age group to account for different testing strategies by age. In addition, we calculated the OR to assess the association between adenovirus positivity and ICU/HDU admission or transplant separately for UK and other countries.

Although previous analyses have indicated that the positivity rate is highest in whole blood [7,13], we analysed adenovirus PCR positivity combining all sample types (whole blood, serum, stool and respiratory, and other/unknown sample type). Percentages of positivity for adenovirus, SARS-CoV-2 and other pathogens were calculated, using as denominator the number of cases tested for each pathogen. The diagnostic method for pathogens other than adenovirus and SARS-CoV-2 was not available in TESSy. Percentages are only reported for pathogens where the test was performed in at least 15% of the cases. For the rest we only present number of positive cases and total cases tested. 

Epidemiological curves were plotted by ISO week based on the date of onset of first symptoms of the disease (available for 60.4% of cases) or, when unavailable, on the date of hospitalisation.

For the country comparison, cases older than 10 years were excluded since there was a substantial difference in the proportion of these cases in the UK compared with other countries (4.2% vs 13.8%).

Data cleaning, preparation and analyses were performed using StataSE 17 (StataCorp, College Station, United States).

## Results

**Figure 1:**
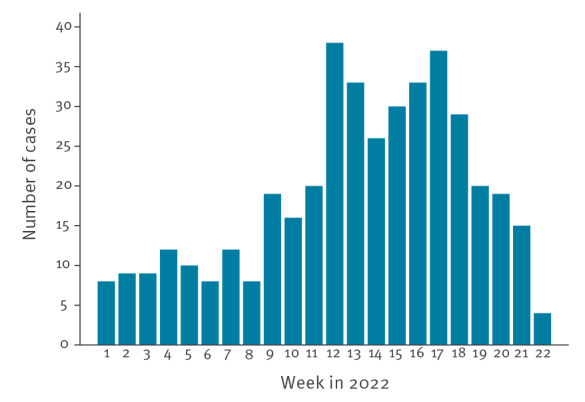
Distribution of cases of severe acute hepatitis of unknown aetiology in children ≤ 16 years-old, by onset of first symptoms or date of hospitalisation^a^, WHO European Region, weeks 1–22, 2022 (n = 427)

A total of 427 cases were reported by 20 countries. More than half of the cases (59.0%) occurred between weeks 9 and 17 of 2022. A decline in the number of cases was observed from week 18 (Figure 1).

WHO: World Health Organization.

^a^ Where date of onset was not available we used date of hospitalisation.

Among cases with at least one test result available for any pathogen, 62.0% (233/376) were positive for at least one, although the number of cases tested for specific pathogens was variable. Adenovirus results from either whole blood, serum, respiratory, faecal or other/unknown sample type were available from 325 cases. Of these, 53.5% (n = 174) had at least one sample with a positive result.

A total of 280 children were tested for SARS-CoV-2 using RT-PCR, of which 10.4% (n = 29) were positive. Results from SARS-CoV-2 serology were available for 47 children, of whom 31 were positive. Of the 117 children that were tested for both adenovirus and SARS-CoV-2, 12 (10.3%) had a coinfection.

Other pathogens were also detected through PCR or serology, presented here as percentage; cases testing positive/cases tested; the exact technique used for each sample was not known to us. Cytomegalovirus (9.0%; 20/223), Epstein-Barr virus (18.7%; 39/209), herpes simplex virus 1 (2.3%; 3/129), respiratory syncytial virus (4.2%; 5/120), human herpesvirus 6 (24.5%; 26/106), parvovirus (2.1%; 2/97), influenza virus (3.6%; 3/83), human herpesvirus 7 (34.2%; 25/73), enterovirus (15.4%; 10/65), varicella-zoster virus (1/58), *Mycoplasma* spp. (2/50), rotavirus (2/22), sapovirus (3/22), norovirus (1/22), bocavirus (1/9), rhinovirus (9/14), antistreptolysin O titer (1/5). Bacterial PCR from stool as well as stool bacterial and viral cultures and throat bacterial cultures were performed in a small number of cases and detected *Escherichia coli* 0157 (1/3), rotavirus (1/3) and *Streptococcus* spp. (1/3).

Of 261 cases with complete information about ICU/HDU admission, 84 (32.2%) were admitted. Of 208 cases with complete information about transplantation, 18 (8.7%) received a liver transplant and two were on the waiting list for transplantation.

### Analysis by age

As shown in Table 1, children aged 0–5 years accounted for the majority of cases (77.3%, n = 330). Children aged 6–10 years and 11–16 years accounted for 14.8% (n = 63) and 8.0% (n = 34) of cases, respectively. The mean age of the cases was 4.2 years and median age was 3 years. Half of the cases were female (52.3%; 201/384) and there was no significant difference in sex distribution between age groups (p = 0.15).

**Table 1:** Infection positivity and clinical outcome by age group for cases of hepatitis of unknown aetiology, WHO European Region, 1 January–16 June 2022 (n = 427)

Age group	0–5 years				6–10 years				11–16 years			
Number of cases per age group	n = 330, 77.3%				n = 63; 14.8%				n = 34; 8.0%			
Outcome	n^a^	N^b^	%	OR^c^ (95% CI)	n^a^	N^b^	%	OR	n^a^	N^b^	%	OR
Adenovirus (any sample)	152	254	59.8	2.32 (1.28–4.21)	21	54	38.9	Ref	1	17	5.9	Nc
Adenovirus (whole blood)	110	178	61.8	3.29 (1.57–6.90)	12	37	32.4	Ref	0	10	0	Nc
SARS-CoV-2 (PCR)	23	220	10.5	1.30 (0.40–4.24)	3	41	7.3	Ref	3	19	15.8	Nc
ICU/HDU	74	204	36.3	1.98 (0.88–4.47)	8	37	21.6	Ref	2	20	10.0	Nc
Transplanted	16	151	10.6	1.68 (0.42–6.69)	2	37	5.4	Ref	0	20	0	Nc

CI: confidence interval; HDU: high dependency unit; ICU: intensive care unit; Nc: not calculated; OR: odds ratio; Ref: reference; SARS-CoV-2: severe acute respiratory syndrome coronavirus 2; WHO: World Health Organization.

^a^ Number of cases with a positive outcome (positive test result, admitted to ICU/HDU or transplanted).

^b^ Total number of cases with complete information on this variable (pathogen or outcome).

^c^ The OR displayed shows the unadjusted OR of a positive test or of having the outcome in the age group 0–5 years compared with the reference group 6–10. We could not estimate OR for the group 11–16 years because of small numbers.

Adenovirus positivity (Table 1) was highest among children aged 0–5 years (59.8%), with an OR of 2.32 (95% CI: 1.28–4.21) compared with the age group 6–10 years. Stratifying the analysis by sample type showed that positivity in whole blood samples was 61.8% in children aged 0–5 years and 32.4% in children aged 6–10 years (OR = 3.29; 95% CI: 1.57–6.90). There was no significant difference in positivity by age in respiratory, faecal or serum samples, although the numbers were small. There was no statistically significant difference in overall adenovirus infection by sex. Regarding SARS-CoV-2 test results, there were no significant differences in PCR and serology positivity rate by age group.

The percentage of cases admitted to ICU/HDU was 36.3% in children aged 0–5 years, 21.6% in those aged 6–10 years and 10.0% in those aged 11 to16 years (Table 1). Sixteen of the 18 transplanted children were aged 0–5 years but there was no significant difference by age group in the odds of transplantation (OR = 1.68; 95% CI: 0.42–6.69).

### Outcome by infection status

Cases with a positive adenovirus result were statistically significantly more likely to be admitted to ICU/HDU (42.1% vs 25.5%, OR = 2.11; 95% CI: 1.18–3.74) and to receive a transplant (16.7% vs 5.3%; OR = 3.36; 95% CI: 1.19–9.55) than cases without infection (Table 2). All cases with a confirmed adenovirus infection who required a liver transplant were aged 0–5 years. Only four cases positive for SARS-CoV-2 were admitted to ICU/HDU and one case required a liver transplant (Table 2). However, overall SARS-CoV-2 positivity was low among cases with a known outcome.

**Table 2:** Outcome by infection positivity for cases of hepatitis of unknown aetiology in the WHO European Region, 2022 (n = 427)

	Adenovirus (all samples)								SARS-CoV-2 (PCR)							
	Positive result				Negative result				Positive result				Negative result			
	n^a^	N^b^	%	OR^c^ (95% CI)	n^a^	N^b^	%	OR	n^a^	N^b^	%	OR^c^ (95% CI)	n^a^	N^b^	%	OR
ICU/HDU	51	121	42.1	2.11 (1.18–3.74)	25	98	25.5	Ref	4	21	19.1	0.35 (0.12–1.02)	71	167	42.5	Ref
Transplant	13	78	16.7	3.36 (1.19–9.55)	5	94	5.3	Ref	1	15	6.7	0.80 (0.14–4.72)	14	119	11.8	Ref

CI: confidence interval; HDU: high dependency unit; ICU: intensive care unit; OR: odds ratio; Ref: reference; SARS-CoV-2: severe acute respiratory syndrome coronavirus 2; WHO: World Health Organization.

^a^ Number of cases with a given test result that were admitted to ICU/HDU or were transplanted.

^b^ Total cases with complete information on the outcome (ICU/HDU admission and transplantation) among cases with a given result for adenovirus (all samples) or SARS-CoV-2 (PCR).

^c^ The OR displayed shows the unadjusted OR of having the outcome among cases testing positive relative to cases testing negative.

Of all cases admitted to ICU/HDU, 26 were positive for at least two pathogens. Of these, two were positive for adenovirus and SARS-CoV-2 and 21 for adenovirus and another pathogen. Among the transplanted cases, five had a co-infection. One case was positive for adenovirus and SARS-CoV2 and the other four cases were positive for adenovirus and at least two other pathogens. However, complete information about testing was not available for all these cases.

### Comparative analysis of cases from the United Kingdom and other countries in the WHO European Region

**Figure 2:**
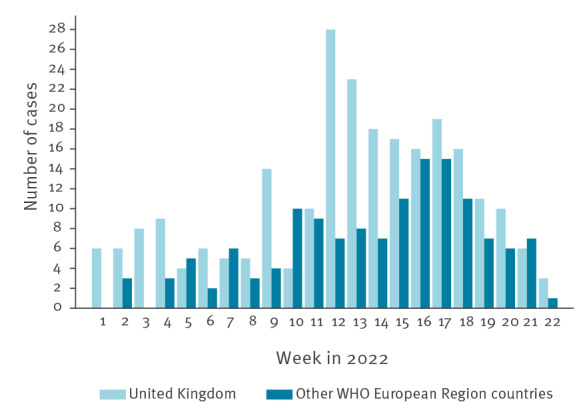
Distribution of cases of severe acute hepatitis of unknown aetiology in children ≤ 10 years-old, by onset of first symptoms or date of hospitalisation^a^, United Kingdom and other WHO European Region countries, weeks 1–22, 2022 (n = 393)

A total of 393 cases 10 years and younger were reported, of whom 63.4% were from the UK. An increase in cases was observed earlier in the UK than in other countries (Figure 2).

WHO: World Health Organization.

^a^ Where date of onset was not available we used date of hospitalisation.

The cases in the UK were younger than in other countries (214/249; 85.9% vs 116/144; 80.6% aged 0–5 years; p = 0.161), although this difference was not statistically significant. Looking at the information available on testing for adenovirus infection, 81.1% (202/249) of cases in the UK and 73.6% (106/144) of cases in other countries had a test result available from any sample type. Among cases with a result available, prevalence of adenovirus infection was 63.6% among the UK cases compared with 35.3% in other countries (p < 0.001). When adjusting by age, cases in the UK were still significantly more likely to have an adenovirus infection than cases reported from other countries (OR = 3.10; 95% CI: 1.90–5.07). In the UK, 65.1% of the cases (162/249) had an adenovirus test result available from whole blood, compared with 36.8% of cases in other countries (53/144). The adenovirus positivity rate in whole blood samples was 63.6% in cases from the UK and 35.9% in cases from other countries, p < 0.001. When adjusted by age, cases in the UK were still significantly more likely to have an adenovirus infection in whole blood samples than cases reported from other countries (OR = 3.10; 95% CI: 1.43–6.52). There was no significant difference between the UK and other countries in the positivity rate for SARS-CoV-2. A statistically significantly higher proportion of cases from the UK was admitted to ICU/HDU (52.9%; 74/140) compared with other European countries (7.9%; 8/101; p < 0.001). When looking at the association between adenovirus positivity and ICU/HDU admission separately between UK and other countries, this association was no longer statistically significant, with an OR of 0.82 (95% CI: 0.38–1.75) in the UK and an OR of 3.49 (95% CI: 0.69–17.62) in other countries. When performing this same analysis for adenovirus positivity and transplantation, the OR among UK cases and cases from other countries were both above 1, but the results were not statistically significant because of the small number of transplant cases in our sample.

## Outbreak control measures

Following the alert from the UK to the WHO on 5 April 2022, the ECDC together with the WHO Regional Office for Europe quickly established a case-based surveillance system to enable the reporting of cases to TESSy from countries within the WHO European Region [12]. The ECDC published a laboratory testing guidance to advise countries on microbiological investigation of the cases and provided support around the molecular characterisation and metagenomic analyses of suspected cases [14]. The WHO has also developed a guidance to provide testing considerations and strategies, together with a suggested minimum set of variables to collect and a case reporting form to support standardised data collection [15-17]. A protocol describing a standardised process to calculate the baseline of cases with severe acute hepatitis of unknown aetiology using ICD-10 or ICD-9 codes was developed. Work is ongoing to estimate whether there is an exceedance of cases compared with this baseline in the European Union and European Economic Area. Finally, work on different potential study protocols is underway to identify the aetiology of the cases reported in this event.

The WHO and ECDC held webinars with countries, research networks and various Centres for Disease Control and Public Health Authorities, which were helpful in order to facilitate exchange of experiences, current knowledge and measures taken. 

## Discussion

In this paper, we describe the characteristics of 427 cases of severe acute hepatitis of unknown aetiology in children in the WHO European Region that were reported to TESSy with date of onset or date of hospitalisation from 1 January 2022 to 16 June 2022. The data show an increase in the number of cases starting in week 12, 2022; this increase was seen earlier in the UK than in other countries in the WHO European Region. It is uncertain whether this reflects a true difference in disease incidence or a detection and reporting bias as the peak of European cases coincides with the week of the beginning of the European surveillance (week 17, 2022). Our analysis indicates a possible decline in cases across the UK and other countries from week 18, 2022. However, as severe hepatitis can develop weeks after the onset of the first symptoms and as case investigations require several days or even months to exclude specific causes, there may be a delay in the reporting and recent trends are challenging to interpret. 

Most of the cases reported were children 5 years or younger. Our analysis suggests that cases in this age group had a higher adenovirus positivity, however, surveillance data of adenovirus circulation shows that this age group is usually more affected than older children and adults [4,7]. Children 5 years or younger also accounted for most of the ICU/HDU admissions and liver transplantations. These findings may indicate a higher likelihood of severe disease in younger children, but it is not clear if reported cases across all ages share the same aetiology. It may also prove difficult to identify a definite causative agent. Previous investigations of adult cases with acute liver failure of unknown origin reported a variety of infectious agents and several cases of concurrent infections with multiple viruses [18].

The characteristics of the cases differed across countries. Cases in the UK were more likely to have tested positive for adenovirus infection and more likely to have been admitted to ICU/HDU than cases from other countries. This may be due to differences in surveillance practices, case reporting, hospitalisation or ICU/HDU admission criteria or differences in adenovirus assays and testing protocols used. We have attempted to minimise these possible biases by only including cases tested for adenovirus and by running separate analyses for adenovirus test results from any sample type vs whole blood samples. There may still be differences in the assays used that could explain some of the differences in positivity. However, the differences between cases in the UK and other countries might also reflect differences in underlying aetiologies. Cases outside the UK may represent the baseline cases of severe acute hepatitis of unknown aetiology affecting a broader age group. The UK has seen both a sharp increase in adenovirus circulation in children younger than 5 years starting at the end of 2021, which can be partially explained by an increase in testing and an excess of hepatitis cases in the same age group [4,7]. In the Netherlands, virological data from the weekly sentinel surveillance system of Dutch medical microbiological laboratories have shown an increase in the number of detections of specific adenovirus types since October 2021 (data not shown). Investigations into adenovirus circulation and work to determine if the number of cases reported are above what would normally be expected are still ongoing across the European Region.

We observed a correlation between adenovirus positivity and disease severity, with a statistically significant association with ICU/HDU admission and liver transplantation. However, this was not statistically significant when performing the analysis separately for the UK and other countries. The fact that an association was seen in the pooled analysis is probably due to the fact that cases in the UK were more likely to test positive for adenovirus because of the ongoing increased circulation of this virus in the population and also more likely to be admitted to ICU/HDU. Assuming that adenovirus is involved in the disease pathogenesis, it is plausible that younger children had been more susceptible to adenovirus infections following the reduced circulation of common respiratory and enteric viruses during the coronavirus disease (COVID-19) pandemic restrictions [19,20]. However, awareness of the event and of the possible role played by adenovirus, or adenovirus associated with a co-factor, might have led to a targeted testing strategy for adenovirus, thus creating a coincidental and not causal association.

This study has several limitations. Although all countries were asked to submit data according to the ECDC/WHO definition, differences remained, particularly in the selection of diagnostic tests performed to exclude other possible causes. At the time of reporting, some children may not have undergone the full diagnostic screening for other possible causes of hepatitis, or some diagnostic tests results were still pending. This may have led to under-reporting or misclassification of some reported cases. Similarly, the difference in the proportion of cases older than 10 years between the UK and other countries may be explained by differences in case definitions.

Data completeness was low on several variables. This can be explained by differences in testing practices and by cases being reported retrospectively, with limited availability of stored samples for further testing. Finally, the UK has reported large variations in detection capabilities of adenovirus assays, thus differences in positivity between countries may be a reflection of assay performance [7].

## Conclusion 

This study provides insights into the characteristics of children with hepatitis of unknown aetiology, including significant differences between cases in the UK and those in other countries, which warrant further investigation. Greater efforts are needed to establish whether the reported cases are above the baseline incidence of severe hepatitis of unknown aetiology in children and to better define this syndrome and standardise the diagnostic algorithms. A continuous collection of detailed clinical, microbiological and epidemiological data on probable cases is therefore important. Well-designed and coordinated analytical studies are necessary to identify risk factors and the aetiological agents involved in this syndrome.

## References

[1] Braccio S Irwin A Riordan A Shingadia D Kelly DA Bansal S Acute infectious hepatitis in hospitalised children: a British Paediatric Surveillance Unit study. Arch Dis Child. 2017;102(7):624-8. 10.1136/archdischild-2016-311916 28377449

[2] Squires RH Jr Shneider BL Bucuvalas J Alonso E Sokol RJ Narkewicz MR Acute liver failure in children: the first 348 patients in the pediatric acute liver failure study group. J Pediatr. 2006;148(5):652-8. 10.1016/j.jpeds.2005.12.051 16737880 PMC2662127

[3] Alonso EM Horslen SP Behrens EM Doo E. Pediatric acute liver failure of undetermined cause: A research workshop. Hepatology. 2017;65(3):1026-37. 10.1002/hep.28944 27862115 PMC5372202

[4] Marsh K Tayler R Pollock L Roy K Lakha F Ho A Investigation into cases of hepatitis of unknown aetiology among young children, Scotland, 1 January 2022 to 12 April 2022. Euro Surveill. 2022;27(15):2200318. 10.2807/1560-7917.ES.2022.27.15.2200318 35426362 PMC9012090

[5] World Health Organization (WHO). Acute hepatitis of unknown aetiology – the United Kingdom of Great Britain and Northern Ireland. Geneva: WHO; 2022. Available from: https://www.who.int/emergencies/disease-outbreak-news/item/2022-DON368

[6] United Kingdom Health Security Agency (UKHSA). Increase in hepatitis (liver inflammation) cases in children – latest updates. London: UKHSA; 2022. Available from: https://www.gov.uk/government/news/increase-in-hepatitis-liver-inflammation-cases-in-children-under-investigation

[7] United Kingdom Health Security Agency (UKHSA). Investigation into acute hepatitis of unknown aetiology in children in England. Technical briefing 3. London: UKHSA; 2022. Available from: https://assets.publishing.service.gov.uk/government/uploads/system/uploads/attachment_data/file/1077027/acute-hepatitis-technical-briefing_3.pdf

[8] United Kingdom Health Security Agency (UKHSA). Investigation into acute hepatitis of unknown aetiology in children in England. Technical briefing. London: UKHSA; 2022. Available from: https://assets.publishing.service.gov.uk/government/uploads/system/uploads/attachment_data/file/1071198/acute-hepatitis-technical-briefing-1_4_.pdf

[9] United Kingdom Health Security Agency (UKHSA). Investigation into acute hepatitis of unknown aetiology in children in England. Technical briefing 2. London: UKHSA; 2022. Available from: https://assets.publishing.service.gov.uk/government/uploads/system/uploads/attachment_data/file/1073704/acute-hepatitis-technical-briefing-2.pdf

[10] de Kleine RH Lexmond WS Buescher G Sturm E Kelly D Lohse AW Severe acute hepatitis and acute liver failure of unknown origin in children: a questionnaire-based study within 34 paediatric liver centres in 22 European countries and Israel, April 2022. Euro Surveill. 2022;27(19):2200369. 10.2807/1560-7917.ES.2022.27.19.2200369 35551705 PMC9101968

[11] van Beek J Fraaij P Giaquinto C Shingadia D Horby P Indolfi G Case numbers of acute hepatitis of unknown aetiology among children in 24 countries up to 18 April 2022 compared to the previous 5 years. Euro Surveill. 2022;27(19):2200370. 10.2807/1560-7917.ES.2022.27.19.2200370 35551703 PMC9101970

[12] European Centre for Disease Prevention and Control (ECDC). Hepatitis of unknown origin - Reporting protocol 2022. Version 2.1. Stockholm: ECDC; 2022. Available from: https://www.ecdc.europa.eu/en/publications-data/hepatitis-unknown-origin-reporting-protocol-2022

[13] European Centre for Disease Prevention and Control (ECDC), World Health Organization Regional Office for Europe (WHO/Europe). Hepatitis of unknown origin in children. Joint Epidemiological overview. Stockholm: ECDC; Copenhagen: WHO/Europe; 2022. Available from: https://www.ecdc.europa.eu/en/hepatitis/joint-weekly-hepatitis-unknown-origin-children-surveillance-bulletin

[14] European Centre for Disease Prevention and Control (ECDC). Guidance for diagnostic testing of cases with severe acute hepatitis of unknown aetiology in children. Stockholm: ECDC; 2022. Available from: https://www.ecdc.europa.eu/en/publications-data/guidance-diagnostic-testing-cases-severe-acute-hepatitis-unknown-aetiology

[15] World Health Organization (WHO). Laboratory testing for severe cute hepatitis of unknown aetiology in children: interim guidance, 17 June 2022. Geneva: WHO; 2022. Available from: https://www.who.int/publications/i/item/who-unkhep-laboratory-2022.1

[16] World Health Organization (WHO). Suggested minimum variables for reporting cases of severe acute hepatitis of unknown aetiology in children: line list, 17 June 2022. Geneva: WHO; 2022. Available from: https://www.who.int/publications/i/item/WHO-UnkHep-Surveillance-Line_list-2022.1

[17] World Health Organization (WHO). The WHO Global Clinical Platform for severe acute hepatitis of unknown aetiology in children. Geneva: WHO; 2022. Available from: https://www.who.int/tools/global-clinical-platform/severe-acute-hepatitis-of-unknown-aetiology-in-children

[18] Somasekar S Lee D Rule J Naccache SN Stone M Busch MP Viral surveillance in serum samples from patients with acute liver failure by metagenomic next-generation sequencing. Clin Infect Dis. 2017;65(9):1477-85. 10.1093/cid/cix596 29020199 PMC5848299

[19] Reicherz F Xu RY Abu-Raya B Majdoubi A Michalski C Golding L Waning immunity against respiratory syncytial virus during the COVID-19 pandemic. J Infect Dis. 2022;jiac192. 10.1093/infdis/jiac192 35524952 PMC9129162

[20] Sanz-Muñoz I Tamames-Gómez S Castrodeza-Sanz J Eiros-Bouza JM de Lejarazu-Leonardo RO. Social distancing, lockdown and the wide use of mask; a magic solution or a double-edged sword for respiratory viruses epidemiology? Vaccines (Basel). 2021;9(6):595. 10.3390/vaccines9060595 34205119 PMC8228489

